# Portrait of a Pathogen: The *Mycobacterium tuberculosis* Proteome *In Vivo*

**DOI:** 10.1371/journal.pone.0013938

**Published:** 2010-11-11

**Authors:** Nicole A. Kruh, Jolynn Troudt, Angelo Izzo, Jessica Prenni, Karen M. Dobos

**Affiliations:** 1 Department of Microbiology, Immunology and Pathology, Colorado State University, Fort Collins, Colorado, United States of America; 2 Proteomics and Metabolomics Facility, Colorado State University, Fort Collins, Colorado, United States of America; 3 Department of Biochemistry and Molecular Biology, Colorado State University, Fort Collins, Colorado, United States of America; Cairo University, Egypt

## Abstract

**Background:**

*Mycobacterium tuberculosis* (*Mtb*), the causative agent of tuberculosis (TB), is a facultative intracellular pathogen that can persist within the host. The bacteria are thought to be in a state of reduced replication and metabolism as part of the chronic lung infection. Many *in vitro* studies have dissected the hypothesized environment within the infected lung, defining the bacterial response to pH, starvation and hypoxia. While these experiments have afforded great insight, the picture remains incomplete. The only way to study the combined effects of these environmental factors and the mycobacterial response is to study the bacterial response *in vivo*.

**Methodology/Principal Findings:**

We used the guinea pig model of tuberculosis to examine the bacterial proteome during the early and chronic stages of disease. Lungs were harvested thirty and ninety days after aerosol challenge with *Mtb*, and analyzed by liquid chromatography-mass spectrometry. To date, *in vivo* proteomics of the tubercle bacillus has not been described and this work has generated the first large-scale shotgun proteomic data set, comprising over 500 unique protein identifications. Cell wall and cell wall processes, and intermediary metabolism and respiration were the two major functional classes of proteins represented in the infected lung. These classes of proteins displayed the greatest heterogeneity indicating important biological processes for establishment of a productive bacterial infection and its persistence. Proteins necessary for adaptation throughout infection, such as nitrate/nitrite reduction were found at both time points. The PE-PPE protein class, while not well characterized, represented the third most abundant category and showed the most consistent expression during the infection.

**Conclusions/Significance:**

Cumulatively, the results of this work may provide the basis for rational drug design – identifying numerous *Mtb* proteins, from essential kinases to products involved in metal regulation and cell wall remodeling, all present throughout the course of infection.

## Introduction

The *Mycobacterium tuberculosis* (*Mtb*) bacillus has the ability to lie dormant in the human body for decades, only progressing to active disease in 5–10% of immunocompetent individuals. The organism is transmitted through aerosols, and enters the pulmonary system through inhalation. Within the lung, the bacillus can take up residence inside an alveolar macrophage triggering the aggregation of immune cells and the formation of a granuloma. During the course of infection, granulomas play a dual role - serving as a niche for the invading bacteria, whilst, protecting the host from active disease. The population of granulomas within the infected host consists of both primary and post-primary lesions. Primary granulomas containing the inhaled founder strain are morphologically different from post-primary granulomas that have developed through disseminated infection. This results in a heterogeneous population of bacilli that are unique to the *in vivo* experience [1], [2], [3].

Substantial research has been dedicated to determining the cellular architecture and molecular features of the host response, including the granulomatous response, its formation and the role of the host response in containing the bacterium. Until recently few studies have focused on the significance of the bacterial contribution within the infected host. Depictions of the mycobacterial proteome during infection thus far have been simulated through *in vitro* studies – utilizing either infected cell culture [4], [5] or through the mimicry of hypoxic environments [6], [7], [8], [9]. Further, models of nutrient starvation [10] and non-replicative persistence (NRP) [11] have also contributed to the overall dissection of the bacterium's intracellular lifestyle. More recently, bioinformatics was used to pool the overwhelming amount of data from these studies, extracting the commonalities and proposing new drug targets and vaccine candidates [12], [13]. Specifically, these studies illustrated the importance of proteins involved in the transport of sulfur and cations, iron scavenging and nitrogen reduction. While experiments reflective of the global gene expression profile of *Mtb* during the in vivo infection provide a more relevant picture of bacterium during infection [14], to date, no thorough proteomic studies have been performed on *in vivo* samples. In order to better understand the bacterial populations within the lung, we believe a proteomic approach is necessary to gain insight into the fundamental physiological state of *Mtb* during infection and the mycobacterial response within the infected host tissue.

Using the guinea pig model of aerosol infection, our study has identified over 500 mycobacterial proteins present over the course of infection. Our *in vivo* data gives solidarity to many of the *in vitro* models of dormancy and is enhanced by the absence of artifacts from *in vitro* growth in culture medium. Together, our results yield a picture of the bacterial expression profile during infection.

## Results and Discussion

### Optimization of sample processing for mass spectrometry

**Figure 1:**
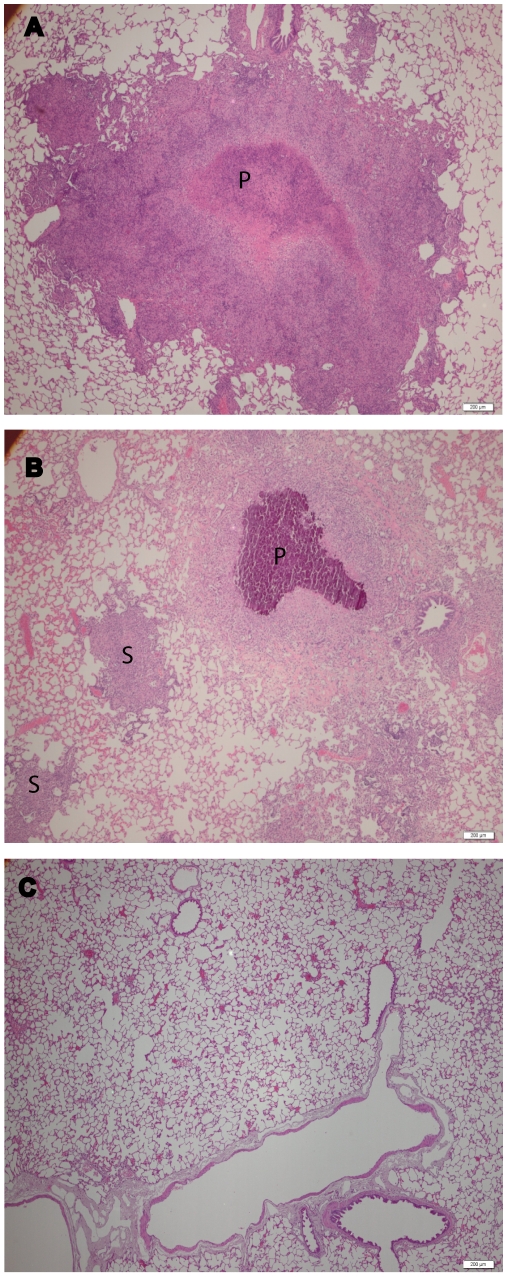
Representative photomicrographs of A) day 30, B) day 90 post-infection guinea pig lungs, and C) uninfected guinea pig lungs. Guinea pigs were aerosolized with a low dose infection of virulent *Mtb* (H37Rv). Tissues were stained (H&E) to demonstrate differences in granulomas based on size (primary granuloma, marked with the letter P) and the absence (secondary granuloma, marked with the letter S) of a necrotic core. Bars are 200 µm.

All *Mtb* protein identifications were derived from the lungs of infected guinea pigs. Since homogenates were made from the whole lung, all proteomic samples contained both host and bacterial proteins. Based on growth curve data from infected guinea pig lungs, 10–20 CFU seeded the lungs of each animal and time-points earlier than 30 days were not addressed due to the challenge of confident protein identification in lung tissue containing less than 5 log_10_ bacilli [15]. The ratio of guinea pig to mycobacterial cells were previously determined using uninfected lung tissue spikes with decreasing numbers of bacteria in order to determine a lower limit of detection with our mass spectrometry methods (data not shown). CFU data was determined for each sample: day 30 samples averaged 5.77 log_10_ (±0.19) and day 90 samples averaged 5.89 log_10_ (±0.32) consistent with previous observations [15]. Similarly, the pathological state of the lungs demonstrated typical progression of chronic tuberculosis, with day 30 infected lungs demonstrating contained lesions consisting of inflammation and areas of central necrosis (Figure 1A). Day 90 infected lungs demonstrated progression of disease with multiple areas of inflammation and coalescing necrosis throughout the lung along with secondary granulomas (Figure 1B) [16], [17]. In either case, a vast majority of each sample was composed of host material. Thus, methodology was developed to significantly reduce host proteins from overwhelming the analyses of *Mtb* proteins. Similar *in vivo* samples previously analyzed by microarray experiments utilized amplification of bacterial RNA or selective analysis of transcripts to eliminate the burden of host RNA. For proteomics, we applied a similar work-flow, whereby chromatographic separation was used to amplify bacterial products via reduction of the sample complexity prior to MS analysis, and construction and interrogation of a smaller custom database (rather than complex databases, ie NCBI or SwissProt) was used for selective analysis of bacterial peptides.

**Figure 2:**
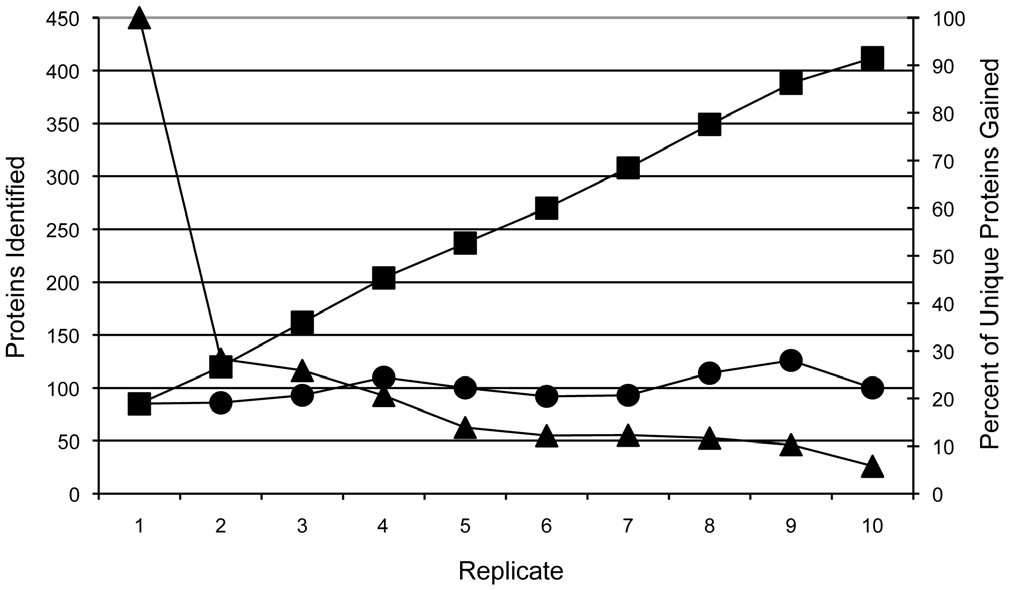
The percent of novel protein identifications taper after sequential injections of MS. Illustration showing the number of proteins identified during 10 replicates (x-axis). The circles signify 50 ng injections of the same WCL digest peptide mixture (standard deviation = 13.1). Squares represent the additive effect of protein identifications after each replicate after combining the Sequest and Mascot result files in Scaffold (left y-axis). The triangles depict the percentage (right y-axis) of unique protein identifications gained per additional replicate.

Initial LC-MS/MS optimization was assessed with tryptic digests of *Mtb* whole cell lysate (WCL) utilizing nanospray mass spectrometry. The number of mycobacterial proteins identified was found to directly correlate to the length of the elution segment during chromatography. A short and shallow gradient (42 min) did not allow for enough of a separation of host and bacterial proteins. Since the host proteins were much more abundant, ion suppression hindered the identification of many bacterial proteins. In this study, multiple gradient conditions were evaluated, and it was determined that with a peptide load of 50 ng, a 90 minute linear gradient provided optimal separation (Table 1). Additionally, the number of unique protein identifications per sample increased drastically with sequential injections of the same sample (Figure 2). Therefore, all samples were run in triplicate. In addition, the database used for interrogation contained the predicted proteins of the *Mtb* H37Rv and mouse proteomes, so that the host ion peaks would not be matched to the predicted m/z for bacterial ions. Data was then subjected to a second interrogation using a reverse database to further eliminate non-mycobacterial specific spectra.

**Table 1:** Summary LTQ method optimization.

Elution time (min)	# of Proteins Identified (standard deviations)
42	43 (8.7)
60	78.7 (0.58)
90	93 (6.7)
120	117.3 (4.7)

Note: A constant amount (50 ng) of peptide was injected, while the elution time (42–120 min) was altered in order to determine the optimal yield per injection (number of injections = 3). The increase in elution time generates better resolution of the complex peptide mixture.

### Composite analysis of *in vivo Mtb* proteomes

**Figure 3:**
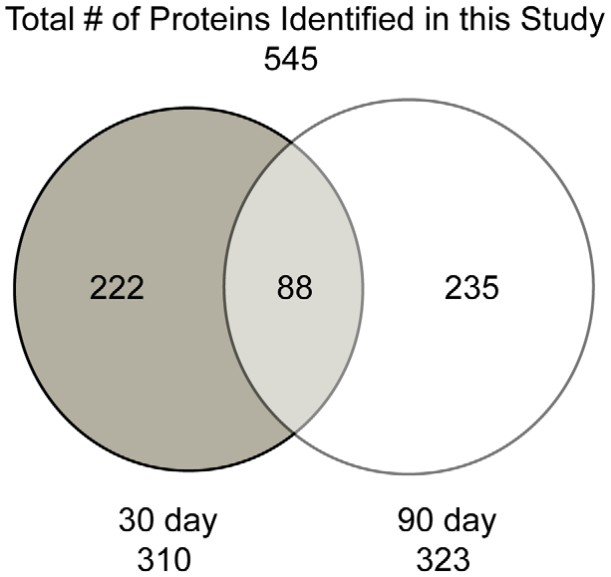
Venn diagram depicting the breakdown of proteins identified at each time-point in this study. While a similar amount of proteins were identified at each of the two infection time points, the overlap is only 28% and 27% of the total identification in the 30 and 90-day samples, respectively.

The sample set consisted of homogenates made from the lungs of six animals, that were harvested 30 and 90 days after low dose aerosol (LDA) infection (total of 12 samples, 6 biological replicates for each time point). The concatenated *Mtb*-mouse database was chosen due to the poor annotation of the guinea pig genome. Since the goal of this study was to identify mycobacterial proteins, rather than host, we felt that the confidence of our protein identifications would be improved by the concatenation to this large mammalian database, which is well-defined and includes over two hundred thousand entries. We are confident in this database due to the homology between the *Mus musculus* and *Cavia porcellus* proteins. Indeed, the most commonly found host proteins, including: albumin, calmodulin, actin, superoxide dismutase, were found whether we used the mouse database or the poorly annotated guinea pig database (data not shown). To reduce the false discovery rate, two separate data filters were designed and applied prior to pooling. The first filter removed proteins identified by peptides that had low ratios of observed to theoretical MS/MS ions – guaranteeing a certain amount of protein coverage and removing bias from larger proteins. The second set of filters was applied at the protein level, retaining only those proteins that were present in 2 or more biological replicates. All filtered data were pooled using the Scaffold program, which added another level of stringency utilizing the Peptide and Protein Prophet statistical analysis algorithms [18], [19]. Proteins and peptides were disqualified below a 90% threshold. Proteins identified by a single peptide were removed from our analysis, while those identified by only two peptides (in separate biological samples) were subject to manual validation. To summarize, from the six 30-day time-point samples, 355,411 spectra were acquired. From these spectra, 1,598 were matched to mycobacterial peptides within 310 proteins. Due to the presence of mammalian tissue, the match ratio was low, accounting for 0.4496% of the spectra. Likewise, from the six 90-day time-point samples, 287,843 spectra were acquired. From these spectra, 2,336 were matched to mycobacterial peptides within 323 proteins, accounting for 0.8116% of the total spectra. This multi-filtered analysis provided a final list of 545 protein identifications, ranging from 10 to 432 kDa with a pI range of 3.54 to 12.12. Between the 30 and 90-day samples, 222 and 235 proteins were uniquely identified and 88 proteins were common between the two sample sets. (Figure 3; Supplementary Tables S1 and S2). As a negative control, six uninfected lung samples were subjected to an identical analysis. Several falsely identified proteins were removed from the final analysis based on the identification of *Mtb* peptides in uninfected that were also found in our infected samples. From this negative control, false discovery rates (FDRs) of 9.1% and 6.9% were calculated in the 30 and 90-day analysis respectively. FDRs were also calculated via the traditional reverse database analysis method and yielded similar FDRs, 11% and 6.8% for 30 and 90-day samples respectively. Since the FDRs calculated from the potential false positives identified in the uninfected samples are similar to the calculated FDRs from the reverse database analysis, a high confidence (90%) for proteins indentified in this analysis was retained.

**Figure 4:**
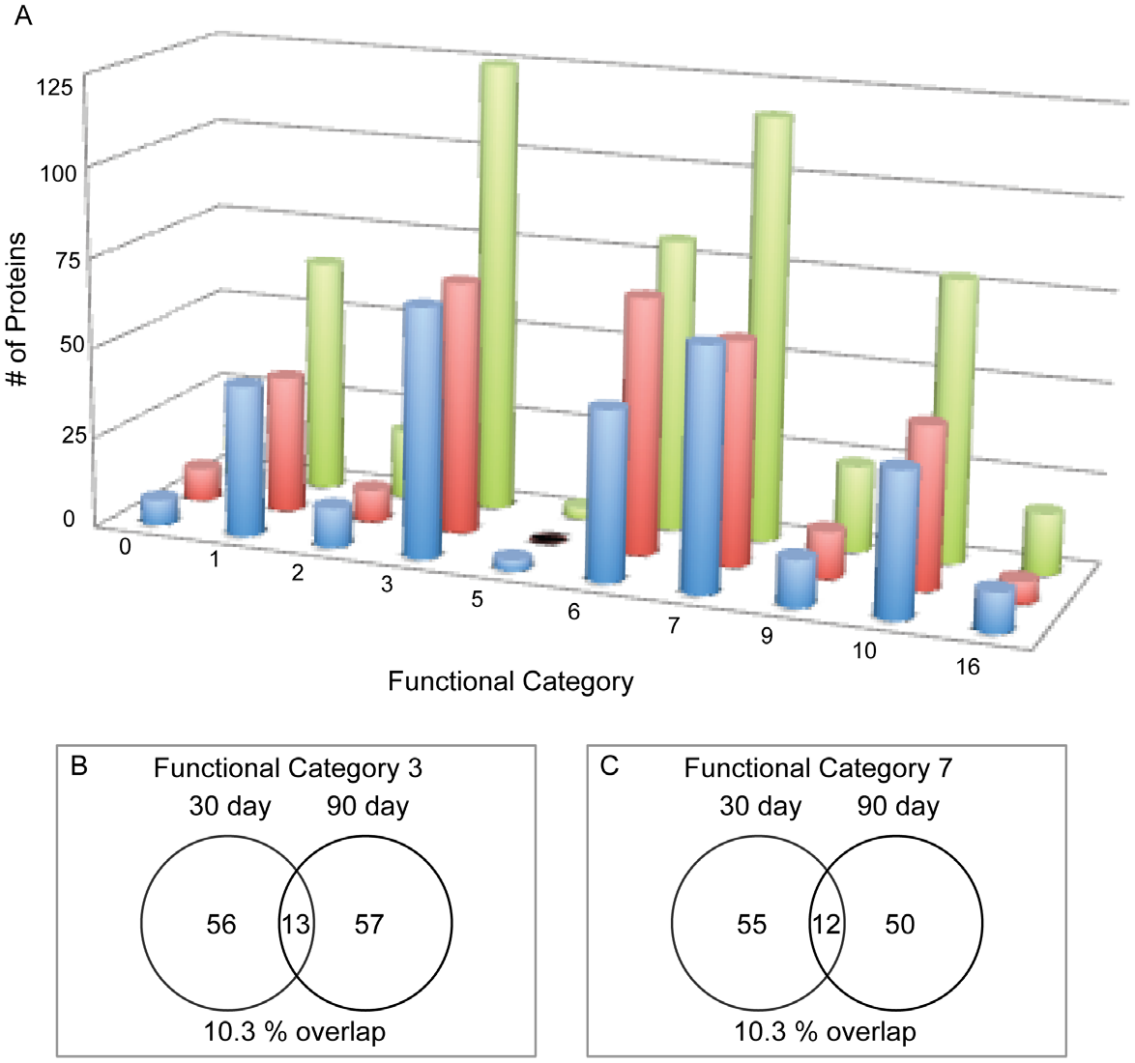
**A**) Comparison of functional categories. The 30-day (blue), 90-day (red) and total identifications (green) were broken down by functional categories. Categories codes are assigned by TubercuList (http://genolist.pasteur.fr/TubercuList/help/function-codes.html): 0 = virulence, detoxification & adaptation; 1 = lipid metabolism; 2 = information pathways; 3 = cell wall & cell processes; 5 = insertion sequences & phages; 6 = PE/PPE; 7 = intermediary metabolism & respiration; 8 = unknown; 9 = regulatory proteins; 10 = conserved hypotheticals and 16 = conserved hypotheticals with an ortholog in *M. bovis*. **B**) Venn diagram showing the overlap between the 30-day and 90-day infection in category 3 and **C**) category 7.

Based on the TubercuList Web Server (http://genolist.pasteur.fr/TubercuList) designations, all proteins were sorted by their functional category (Figure 4A). Two functional groups, categories 3 (cell wall and cell processes) and 7 (intermediary metabolism and respiration) comprised about half of the total data, representing 22.7% and 21% of the total identifications respectively. Interestingly, there is little overlap between the day 30 and day 90 proteins identified for these two categories, with only 13 identical proteins (10.3%) and 12 identical proteins (10.3%) found in both day 30 and day 90 samples (Figure 4B and C).

**Figure 5:**
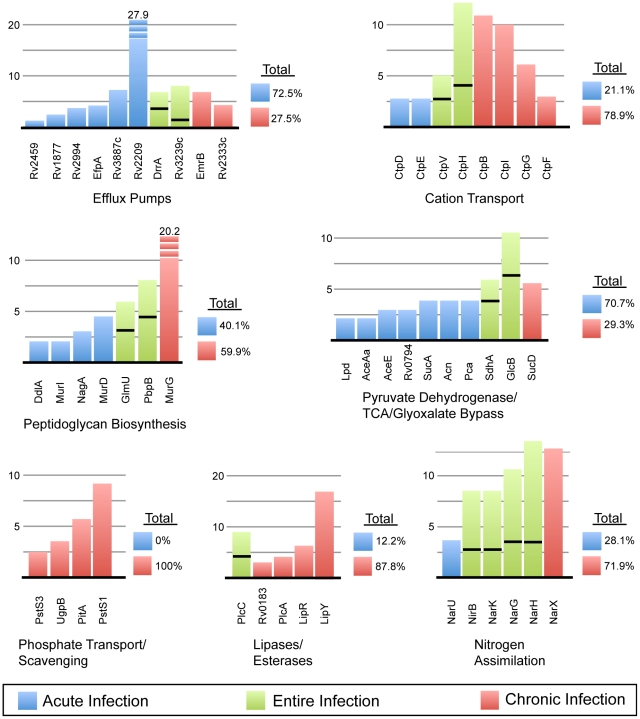
Changes over the course of infection of representative pathways from categories 3 & 7. Each bar in the graph corresponds to the normalized spectral count for each protein within the same category. Each color signifies the infection time-point: blue = 30-day, red = 90-day and green = proteins found at both time-points, of which the area below the black line is the 30-day count and above equals the 90-day count. To the right of each category is the percent breakdown of the total spectral counts in the 30 versus 90-day samples.

A closer look at category 3 shows an abundance of membrane transport proteins (Figure 5). This includes many members of the metal-cation transporting ATPase family, including CtpV and CtpB (copper), CtpD (possibly cadmium), CtpE (unknown), CtpF (unknown), CtpG (unknown), CtbH (unknown) and CtpI (magnesium) – illustrating that the adjustment of cation levels is critical within the host. Just upstream of CtpB (Rv0103), Rv0102 also shows homology (by BLAST analysis) to copper resistance transporters. In addition, 10 efflux pumps were identified and were found to be particularly prevalent in the early stages of infection. Conversely, proteins involved in the binding and transport of phosphate were found entirely in the 90-day data set. The presence and wide variety of pumps and transport proteins during infection lends to the conjecture that the bacteria may adapt to the host environment by scavenging resources and altering the micronutrient levels.

Category 7 includes an assortment of proteins involved in catabolism. While examination of the data demonstrated no significant difference in hexose metabolism between day 30 and 90, significant differences were found in the later stages metabolism, beginning with dehydrogenation of pyruvate through the TCA cycle. Of the 10 proteins identified for this pathway, only 1 was specific to the 90-day samples and the spectra of the remaining 9 proteins are overwhelmingly found in the 30-day samples (Figure 5). This data supports that of others' and indicates a decrease in the preferred carbon nutrients, as well as a decrease in phosphate during the chronic infection [20]. This also supports the hypothesis that mycobacteria may breakdown lipids, rather than carbohydrates, as a source of carbon and energy [21], [22]. The phospholipases C are noted virulence factors and are hypothesized to breakdown host phospholipids for bacterial use [23]. Similarly, Rv0183 – lysophospholipase, LipR (Rv3084) - a lipolytic esterase and LipY (Rv3097) – a triacylglycerol lipase, were all expressed at the 90-day time point. To obtain phosphorous for energy from the host environment, *Mtb* may utilize two types of transport proteins, a low-affinity, PitA and two high-affinity, PstS1 and PstS3 phosphate transporters [24], as well as a phosphate binding lipoprotein. These were present during the chronic infection.

The third most abundant category consists of the acidic PE/PPE proteins, which represent 16.2% of the total protein identifications. It is important to note that the PE/PPE proteins have numerous peptides that overlap or are highly similar. Thus any ambiguities in the assignment of a PE/PPE peptide were analyzed and validated. In this study, all peptides assigned to more than one protein were only retained if the protein in question had an additional two or more unique peptides. Unlike the categories discussed above, this category contains the largest overlap - 37 proteins (45.7%), common between the 30 and 90-day samples. However, from day 30 to day 90, there is also an increase of 7.4% in respect to total proteins represented. This is the largest increase in any of the categories. This increase is most evident in Tables 2 and 3, which show that of the ten most abundant proteins in each sample set by spectral counting, two and five (30 and 90-day, respectively) of these proteins are from the PE/PPE category. The exact role of these proteins is unknown, but in general, these proteins are thought to reside in the cell envelope and have been implicated in increasing antigenic variation [25]. Many PE/PPE proteins, such as PE-PGRS54, have been shown to be upregulated in response to conditions such as hypoxia, exposure to H_2_O_2_ and during NPR [26]. Of interest, it has been hypothesized that some PE and PPE proteins may interact with each other after co-expression from the same operon [27]. Following this notion, two sets of proteins, PE-PGRS53/54 and PE-PGRS56/57, all highly represented in the 90-day infection set, may be products of the same operons.

**Table 2:** The ten most dominant *Mtb* proteins within the 30-day *Mtb*-infected lung samples based on normalized spectral counts.

	Rv#	Protein Name		Normalized Spectral Count		Functional Category	
				30 Day	90 Day	#	Type
1	Rv2209	Rv2209	Probable Conserved Integral Membrane Protein	27.9	0	3	Cell Wall & Cell Processes
2	Rv2315c	Rv2315c	Hypothetical Protein Rv2315c	27	23.5	10	Conserved Hypotheticals
3	Rv0860	FadB	Probable Fatty Oxidation Protein	27	5.6	1	Lipid Metabolism
4	Rv0101	Nrp	Probable Peptide Synthase	22.5	0	1	Lipid Metabolism
5	Rv3512	PE_PGRS56	PE_PGRS Family Protein	20.7	25.8	6	PE/PPE
6	Rv1360	Rv1360	Probable Oxidoreductase	18.9	11.2	7	Intermediary Metabolism & Respiration
7	Rv0015c	PknA	Transmembrane Serine/Threonine-Protein Kinase A	15.3	5.6	9	Regulatory Proteins
8	Rv3403c	Rv3403c	Hypothetical Protein Rv3403c	15.3	0	16	Conserved Hypotheticals with an Orthologue in *M. bovis*
9	Rv3507	PE_PGRS53	PE_PGRS Family Protein	14.4	68.3	6	PE/PPE
10	Rv3859c	GltB	Probable Ferredoxin-Dependent Glutamate Synthase	13.5	8.9	7	Intermediary Metabolism & Respiration

Note: Early in the infection there are many functional categories represented. Many of the proteins are transient, having lower spectral counts or completely absent at day 90. The major exceptions to this are the PE_PGRS proteins and the hypothetical protein Rv2315c.

**Table 3:** The ten most dominant *Mtb* proteins within the 90-day *Mtb*-infected lung samples based on normalized spectral counts.

	RV#	Protein Name		Normalized Spectral Count		Functional Category	
				30 Day	90 Day	#	Type
1	Rv2244	AcpM	Acyl Carrier Protein	0	118.7	1	Lipid Metabolism
2	Rv3507	PE_PGRS53	PE_PGRS Family Protein	14.4	68.3	6	PE/PPE
3	Rv2352c	PPE38	PPE Family Protein	0	48.2	6	PE/PPE
4	Rv3447c	Rv3447c	Probable Conserved Membrane Protein	9.9	39.2	3	Cell Wall & Cell Processes
5	Rv2589	GabT	4-Aminobutyrate Aminotransferase	0	31.4	7	Intermediary Metabolism & Respiration
6	Rv3508	PE_PGRS54	PE_PGRS Family Protein	0	31.4	6	PE/PPE
7	Rv2567	Rv2567	Conserved Hypothetical Alanine & Leucine Rich Protein	2.7	30.2	10	Conserved Hypotheticals
8	Rv3514	PE_PGRS57	PE_PGRS Family Protein	8.1	29.1	6	PE/PPE
9	Rv3512	PE_PGRS56	PE_PGRS Family Protein	20.7	25.8	7	Intermediary Metabolism & Respiration
10	Rv2315c	Rv2315c	Hypothetical Protein Rv2315c	27	23.5	10	Conserved Hypotheticals

Note: The chronic state of infection is dominated by an abundance of proteins from the PE/PPE category.

### Other dominant protein groups present in the *in vivo* samples

#### Polyketide Synthesis and Virulence Lipids

The *Mtb* genome encodes a variety of polyketide synthases (*pks*). These large multi-domain proteins carry out a series of complex reactions to construct complex lipids and metabolites. A total of 9 Pks proteins were identified, including Pks4-9, 13, 15 and 17. Four of the five PapAs or polyketide synthase associated proteins were also identified. These proteins are co-regulated with the *pks* genes and the *mmpLs*. Nine of the twelve MmpLs, large membrane-spanning proteins involved in the translocation of lipids, were found in this analysis, consistent with others' hypotheses of their role during infection and their active involvement in virulence [28], [29]. One such secreted virulence lipid, phthiocerol dimycocerosate (PDIM), has been implicated in infection in mice, and more recently, genes involved in PDIM synthesis have been shown as up-regulated in response to lung surfactants [30], [31]. While microarray data show these genes to be up-regulated 2 hour after exposure to lung surfactants, the data presented here shows that many of these proteins, including PpsA-D, Mas, PapA5, DrrA and MmpL7, are present throughout *in vivo* growth. This is also contrary to assumptions made with starvation models, in which many of these genes were shown to be down-regulated [10]. Similarly, sulfolipids (SL) have also been implicated to play a role in virulence and at day 90, MmpL8 and PapA1 were found to be present [32], [33].

#### Cell Wall and Fatty Acid Biosynthesis

The mycobacterial cell envelope is a thick, complex structure composed of covalently linked mycolic acids, arbinogalactan and peptidoglycan [34]. The production of mycolic acids (C70–C90) is the result of the two types of fatty acid synthesis: de novo synthesis of short chain fatty acids by the Fas protein and long chain fatty acid elongation of FAS products by the FASII pathway. Unlike the Fas protein, which like the pks proteins, is a large multifunctional protein, the FASII pathway is composed of individual proteins working in complex to carry out reactions such as condensation, keto-reduction and dehydration. While the Fas protein was found exclusively in the 30-day samples, the members of the FASII pathway were found primarily in the 90-day samples. The FASII pathway forms the long-chain fatty acyl precursors to mycolic acids. One might speculate that the Fas products (C16–C26) are shuttled into pathways, such as PDIM and SL biosynthesis, which are required for virulence and *in vivo* survival. Interestingly, the majority of the proteins involved in the synthesis of peptidogylcan were found in the 30-day lung tissue and none of the arabinogalactan biosynthesis proteins were detected. Only the MurG protein showed a significant presence during chronic infection (Figure 5). This may be indicative of MurG having dual functions, first as the well-characterized last step of peptidoglycan synthesis on the cytoplasmic side of the bacterial membrane during the early infection and an additional role in cell elongation, resulting in filamentous bacilli during the chronic stage [35], [36]. While some residual replication or cell wall formation may be occurring during infection, the cessation of mycolic acid assembly early in infection is consistent with evidence that the bacteria can lose its acid-fast property within the host [37]. This entertains the hypothesis that the resumption of the FASII pathway in the 90-day samples is indicative of the synthesis of free mycolic acids, perhaps to create drug-tolerant persistent bacterial populations [38].

#### Metal Regulation

The impermeable mycobacterial cell wall serves as a barrier to the import/export of micronutrients essential for the catalytic activity for many enzymes. The production and transport of mycobactin, either to the cell membrane or beyond, is critical in the acquisition of host-derived iron. Production of this polyketide siderophore and its ability to scavenge and chelate iron is an essential part of intra-host survival for the bacillus [39], [40], [41]. Enzymes within the mycobactin synthesis pathway MbtA, B, C and D were represented during either day 30 or at both time-points. Likewise, copper is highly toxic but entirely essential for maintaining activity of several mycobacterial enzymes. Therefore its levels must be tightly regulated, using mechanisms such as active export by the membrane protein, CtpV [42], [43]. Similarly, several other cation-transporting atpases were identified in this study, as previously mentioned in the cell wall and cell wall processes section. Due to the prevalence of the Ctp membrane proteins in the infected lung samples, their functions may be investigated further as potential drug targets. In support of this, *ctpG* mutants were attenuated for virulence in the guinea pig, supporting their role in this model [44]. CtpH and CtpV are found at both infection time-points.

#### Protein Kinases and Nitrogen Assimilation

Of the eleven serine/threonine-protein kinases (Pkns) found in the *Mtb* genome, we found eight in the *in vivo* analysis, only three of which (PknA, B and G) are noted to be essential proteins [45], [46]. The inhibition of protein kinases as part of drug therapy has already been noted; as they have shown great utility in other fields, such as in the treatment of certain cancers [47], [48]. PknH is hypothesized to be necessary for adapting to nitrite stress and development of the chronic infection in the murine model [49]. This data corroborates with the presence of this PknH exclusively at the 90-day infection time point. Nitric oxide, generated by macrophages is a rich source of nitrogen, which can be metabolised for incorporation into various bacterial macromolecules. Proteins involved in nitrate/nitrite transport, such as *NarX* have been previously identified in a hypoxia model by microarray analysis (along with *narK2*), as well as by *in situ* hybridization in human lungs [50], [51]. Our proteomic analysis shows the presence of not only the NarX protein, but in addition, 4 of the 6 of the proteins from the nitrate reductase family from the *narGHJI* and *narK2X* operons, consistent with their role in microaerophilic conditions [7], [51], [52]. The *narU* gene product, which plays a role in nitrite excretion, was also found, as was the NirB, nitrite detoxification protein. While this protein class is present throughout infection, an increase in of most of these proteins during the chronic infection may be due to the effects of hypoxia (Figure 5).

### Possible relationships between proteomic and microarray datasets

The proteomic analysis of *Mtb* infected guinea pig lungs 30 and 90-days post-aerosol challenge provides a description of proteins present during the establishment and maintenance of infection. Prior to this study, the majority of the information known about the bacterial state during hypoxia or infection was gleaned from correlative microarray studies. It has been noted that due to post-transcriptional events, the relationship between the amount of mRNA and protein is not 1∶1 [53]. This was very evident when comparing the 30 and 90-day proteomic data to that of the gene expression data sets from the well-characterized *in vitro* models of NRP and starvation. For example, the Muttucumaru et al. data set, which summarizes the changes cells undergo in the transition from aerobic growth to that of NRP1 (microaerophilic) and NRP2 (anaerobic) stage, showed a 6.8% overlap between the genes upregulated in NRP1/2 and our 30/90-day *in vivo* analysis (Table 4) [54]. The commonalities include: *narK2* and *narH* (nitrate reductase), *ppsB* and *mas* (PDIM synthesis), *ctpV* (copper transport) *mbtB* (mycobactin synthesis) and several *ppe* genes. Similar trends in upregulation were apparent in the Voskuil et al microarray analysis of the stationary phase and NRP models, the Rachman et al microarray analysis of *Mtb* in infected lungs and even the Cho et al ICAT study on *in vitro* NRP (Table 4) [8], [11], [55]. While Cho's study identified representatives from important up-regulated pathways, the proteomics methodology employed in our study recognized several more members of each of these pathways – reinforcing their importance *in vivo*. In addition to those similarities, major fundamental differences exist between microarray data sets from *in vitro* studies and the proteomic data described in this study. Specifically, a large amount of chaperones and detoxification proteins were identified in the *in vitro* models. In fact, the single most common finding in the *in vitro* scenarios, including both Rachman and Cho studies, is the upregulation of *hspX* (Rv2031) [11], [55]. It is very likely that HspX is present *in vivo* – however, based on our findings, it is either rapidly exported from the lung or its mass spectra are obscured in our study.

**Table 4:** Overlap of our study with a sampling of *in vitro* studies.

			Present in 30-day (% of 30-day total)	Present in 90-day (% of 90-day total)	Present in both (% of ‘both’ total)	Present in either (% of ‘either’ total)
This Study	30-day		310(100%)	-	-	310(57.2%)
	90-day		-	323(100%)	-	323(60.1%)
	Total		-	-	88(100%)	545(100%)
Rachman[55]		Total = 188	12(3.9%)	16(5.0%)	6(6.8%)	22(4.0%)
Muttucumaru[54]	NRP1 & NRP2	Total = 299	27(8.7%)	21(6.5%)	10(11.4%)	38(7.0%)
Voskuil[8]	Stationary Phase & Low Oxygen	Total = 116	5(1.6%)	9(2.8%)	4(4.5%)	9(1.7%)
Rohde[62]	Macrophage Infection - 24 hours	Total = 115	5(1.6%)	8(2.5%)	2(2.3%)	11(2.0%)
Cho[11]	NRP1 - ICAT	Total = 87	8(2.6%)	8(2.5%)	1(1.0%)	15(2.8%)
	NRP2 - ICAT	Total = 111	19(6.1%)	14(4.3%)	4(4.5%)	29(5.3%)
Betts[10]	Starvation Model - Up	Total = 44	1(0.3%)	1(0.3%)	0(0.0%)	2(0.4%)
	Starvation Model - Down	Total = 70	7(2.3%)	7(2.2%)	5(5.7%)	9(1.7%)

Note: For the most part, *in vitro* studies have less than 5% similarity with the proteomic data in this study. The two studies depicting changes found during NRP1/2 by Muttucumaru and Cho show the highest similarity to this study.

Interestingly, the starvation model [10] appears the least similar to the *in vivo* results described in this study. In fact, the results appear to be reversed – the profile of genes found down regulated in response to starvation are more similar to our infection model than those found to be up-regulated. Perhaps in the lung of the host, the bacteria are not nutrient restricted at all. Since the sequencing of the *Mtb* genome in 1998, it has been known that *Mtb* contains an unusually large number of proteins involved in lipid metabolism [25]. Many FadD and FadE proteins are present in the *in vivo* data set, thus, it is likely that the bacteria are able to breakdown host lipids in order to utilize them as nutrients [56]. Contrary to our results, the Betts' model shows PDIM synthesis to be decreased, as well as down-regulation of several genes, including: *glcB*, *pknB*, *phoS1* and *fas* – many of which have been shown to be present in other infection models [30], [57], [58]. Rv3403, a hypothetical protein found to be in our preliminary 10 most abundant proteins list at day 30 (Table 2), is shown to be down-regulated in the starvation model [10]. However, by day 90 this protein is completely absent and may be potentially related to the decrease in a nutrient in the lung.

Much of the literature defining the bacterial state during NRP has been based on microarray studies, in which changes in mRNA levels in response to the implementation of stresses on cultured *Mtb* were monitored. While it is important to tease out which pathways may be upregulated due to each stress the bacteria faces in the host, it is equally as important to establish the combined and actual effects of intra-host pressure. In this study, none of the simulated *in vitro* (model) environments accurately reflect the protein profile within the lung – even the “multiple stress dormancy model” [59]. This is not entirely surprising; *in vitro* cultures include a different set of variables resulting in bacterial stress, such as nutrients are static and can be exhausted, toxic bacterial byproducts can build up, and physical space is limiting. Likewise, even tissue culture experiments yield an incomplete picture, in that they are missing more complex immunological influences. Perhaps the most important difference is that *in vitro* studies focus on a clonal population, while in the *in vivo* experience different populations exist – a major contributing factor that has hindered the progress of treatment. In order to tease out which proteins vary across the various bacterial populations and host environments, in future studies, primary and secondary granulomas will be compared to uninvolved tissue.

Lastly, we acknowledge that our described *in vivo* proteome lacks the identification of the major secreted proteins. This was unexpected, since in all previous *in vitro* analyses by ourselves and others [6], [60], [61], secreted/stress response proteins such as GroEL, HspX and DnaK, were highly abundant. Similarly, during the development of our mass spectrometry methodology, we utilized uninfected lung tissue spiked with 10^6^ gamma-irradiated (dead) *Mtb* and in this analysis the secreted proteins and several chaperones remained dominant (data not shown). Likewise, microarray analysis of *Mtb* from macrophage culture, show transcripts of the stress-associated proteins in high abundance [62]. Clearly, there is a difference between mock-infected lung tissue, cell culture-based analyses, and lung tissue obtained from an actual infection. Since our proteomics was performed on whole lung homogenates, it is likely that the exported proteins were not identified because these proteins are not simply secreted by the bacillus and retained at the site of infection; rather we hypothesize that these proteins are trafficked to the draining lymph node, serving as important T-cell antigens. These secreted proteins, therefore, may not be valid drug targets because they are not directly associated with the site of infection. However, their role as potential biomarkers, diagnostic reagents, or vaccine candidates remains. In addition to phagocytosis-mediated export of secreted proteins from the lung, some proteins may directly traffic to the blood, sputum, or other bodily fluids. Indeed, members of the antigen-85 complex (Rv3804c, Rv1886c and Rv0129c) have been detected in serum and cerebral spinal fluid [63], [64]. Further, some secreted mycobacterial products may be shuttled away in exosomes; as is the case described for the 19 kDa liproprotein (Rv3763) and lipoarabinomannan (LAM) [65].

### Final remarks

In this study, over 500 *Mtb* proteins present at 30 and 90 days post infection are described. This description provides a picture of the *Mtb* proteome in mammalian lungs. The exclusion of a protein from either sample list does not rule out its presence. These samples are highly complex, containing both host and bacterial peptides. Thus it is likely that only the most dominant *Mtb* proteins (where dominance is reflective by quantity and capacity for mass spectrometry detection based on the physiochemical ionization properties of the protein) are described here. One of the flaws of large-scale shotgun proteomic studies is that dominant peptides can skew the results in a manner that causes some proteins to remain undetected. Thus, tables 3 and 4 contain the proteins observed based on the abundance of unique and repetitively sequenced peptides from one protein. While this is an accepted method to glean which proteins are more dominant relative to other proteins identified in a given sample, their absolute abundance has not been validated. Most of the spectral counts (the spectral counts for each protein identified in this study can be found in Supplementary Table S3.) mentioned in our study are significantly lower (ten-fold) than the dominant host proteins, which are in the hundreds (data not shown). This is entirely expected and is reflective of the nature of the sample – the infected lung contains few bacteria in relation to host cells. Validation of any of the proteins or pathways in question can be performed with a quantitative mass spectrometry technique, like multiple reaction monitoring (MRM); these studies are currently being developed in our laboratory to validate some of our findings.

This proteomic study on infected lung homogenates has supplied a long list of mycobacterial proteins that are present during infection. The most challenging facet to treating tuberculosis is that there are multiple populations of *Mtb* within the lung. Thus, it is not surprising that our data does not correlate with any one *in vitro* model. The *Mtb*-infected lung comprises a heterogeneous and dynamic population of bacteria. One additional source of heterogeneity is the contribution of organisms that have infiltrated from other infection sites, such as the spleen. It is believed that after seeding the spleen, organisms can re-enter the lung as secondary infection sites [2]. Therefore, it is highly likely that these organisms are also sampled in our 90-day post-infection analysis, in addition to those retained in the lung throughout the course of infection. This aspect is beneficial when defining drug targets. We believe that several of the proteins identified in our analysis will give us clues to which pathways and biosynthetic processes of *Mtb* might be worth targeting during an infection. It is difficult to determine which, of the hundreds of proteins identified are important from this study. The comparisons of our dataset to others' afford some conjecture (Table 4). Few studies have looked at specific factors contributing to *Mtb* survival in the guinea pig model of infection. One study did explore survival rates of attenuated *Mtb* and identified 18 mutants with reduced fitness in the guinea pig [44]. Two of the eighteen gene products, Rv1798 and ctpG were identified in this study. As part of our future undertakings, we hope to further mine which of these aspects of *Mtb* physiology persist in specific *Mtb* lesions through continued comprehensive and targeted proteomic profiling during infection in an effort to define novel, specific targets that lead to advances in vaccine and drug discovery efforts.

## Materials and Methods

### *Mtb* samples and cultures for guinea pig infection

*M. tuberculosis* H37Rv whole cell lysate was provided through the NIAID contract “Tuberculosis research materials and vaccine testing (HHSN266200400091c). For guinea pig infections, *Mtb* H37Rv was initially grown for three passages as a pellicle on Proskauer & Beck media (5 g KH_2_PO_4_, 5 g asparagine, 0.6 g MgSO_4_.7H_2_O, 2.5 g magnesium citrate, 20 mL glycerol, 1 L water, pH 7.8) medium to produce seed stocks. Working stocks with a maximum of six passages were expanded from the seed stocks in P&B medium with 0.1% Tween^-^80. Working stocks were prepared at the mid-log phase, and aliquots were stored at –80°C.

### Low dose aerosol (LDA) infection of guinea pig

Out-bred female Hartley guinea pigs weighing 450–500 grams were purchased from Charles River Laboratories (Wilmington, MA). Guinea pigs were maintained under ABSL-3 barrier conditions in isolator cages (Thoren, Hazleton PA). Bacterial suspensions described above were used to infect 12 guinea pigs with H37Rv by the LDA method (approximately 10 CFU) with a Madison Chamber [66]. In addition, a small aliquot of the suspension is plated on 7H11 media to confirm dose of aerosolized bacilli used in each infection. After infection, individuals in each group are weighted weekly and checked daily for signs of disease. The Karnofsky scale for pain and distress was used to evaluate the well being of each guinea pig. Guinea pigs with a score of 8 or more were euthanized. CFUs were determined by plating organ homogenates onto nutrient 7H11 agar supplemented with OADC. Colonies were enumerated after 21-days incubation at 37°C. All animals were handled in strict accordance with good animal practice as defined by the relevant national and/or local animal welfare bodies, and all animal work was approved by the appropriate committee (IACUC #06-239A). The aforementioned experimental procedures were approved by the Colorado State University Institutional Animal Care and Use Committee.

### Preparation of guinea pig lung homogenates

At the 30 or 90-day post-infection time point, guinea pigs were sacrificed and the right cranial lung lobe was used for bacilli counts. The remaining lobes were added to 3 mL of PBS and subject to 4 rounds of homogenization with an Omni MultiMix 200. Samples were removed and homogenization tubes were rinsed with an additional 1 mL of PBS. The total homogenate was then treated with collagenase H (final concentration: 0.7 mg/mL) and DNase (final concentration: 30 µg/ml) for 1 hour at 37°C, shaking. Homogenates were delipidated with 2 rounds of chloroform:methanol (1∶1). The remaining cell pellets were sonicated in PBS and the product was centrifuged at 14k rpm for 10 minutes to remove insoluble particles. The supernatant was mixed with collagen-agarose to remove any residual collagenase. The remaining protein was quantified by bicinchoninic acid (BCA) assay (Pierce). As a control, 6 uninfected guinea pigs were sacrificed on the same day as the 30-day infected guinea pigs. The lungs were processed identically to the infected lungs.

### Processing of proteins isolated from lung tissue

10 µg of each sample lysate was denatured in 6 M guanidine hydrochloride, reduced with 10 mM DTT and alkylated with 100 mM iodoacetamide. After overnight micro-dialysis in 10 mM ammonium bicarbonate the samples were digested with trypsin (1∶50, trypsin:lysate) in 10% acetonitrile (ACN) overnight at 37°C. Digests were dried and resuspended in LTQ loading buffer, 3% ACN and 0.1% formic acid. All digests are performed in deplasticized tubes to reduce plastic polymer contamination.

### Mass Spectrometry of tryptic peptides

All samples were injected at a concentration of approximately 50 – 100 ng/µL. Peptides were purified and concentrated using an on-line enrichment column (Agilent Zorbax C18, 5 µm, 5×0.3 µm column, Agilent 1100 nanoHPLC,). Subsequent chromatographic separation was performed on a reverse phase nanospray column (Zorbax C18, 5 µm, 75 µm ID × 150 mm column). Samples were eluted into a LTQ linear ion trap (Thermo Scientific) using a flow rate of 300 nL/min with the following gradient profile: 0% B for 0–5 min, 0–15% B for 5–8 min, 15–55% B for 8–98 min, and 55–100% B for 98–103 min (A = 3% ACN, 0.1% formic acid; B = 100% ACN, 0.1% formic acid). This elongated method has been optimized to separate complex samples, such as whole cell lysate. Mass spectra are collected over a m/z range of 200–2000 Da using a dynamic exclusion limit of 2 MS/MS spectra of a given mass for 30 s (exclusion duration of 90 s). Compound lists of the resulting spectra were generated using Bioworks 3.0 software (Thermo Scientific) with an intensity threshold of 5,000 and 1 scan/group. All samples were run in triplicate.

### Database searching

All tandem mass spectra (.raw files) were extracted by LCQ_DTA.exe (Thermo Scientific) for subsequent loading into the Mascot (Matrix Science, London, UK; version: 2.1 Mascot) [67] MS/MS search engine or into Bioworks Browser (version 3.3.1 SP1) for subsequent analysis with Sequest (Thermo Finnigan, San Jose, CA; version SRF v.5). Mascot was set up to search a customized database (created on 05/06/08) composed of the *Mycobacterium tuberculosis* H37Rv FASTA (GenBank accession number AL123456, release R9, 3,998 protein genes) concatenated to the IPI Mouse database (International Protein Index - http://www.ebi.ac.uk/IPI/IPIhelp.html), which contains 222,498 entries. Sequest and X!Tandem (Version 2007.01.01.1) were set up to search only the *Mycobacterium tuberculosis* H37Rv database listed above. All searches were performed assuming trypsin digestion, with a fragment ion mass tolerance of 1.00 Da, a parent ion tolerance of 3.00 Da. Mascot and Sequest searches allowed for 1 and 5 missed cleavages, respectively. Oxidation of methionine (+16) and the iodoacetamide derivative of cysteine (+57) were specified as variable modifications.

### Criteria for Protein Identification

Sequest result files were subject to two filters. At the peptide level, peptides were excluded if the ratio of observed to theoretical peptides was <0.2, when the theoretical was less than 100, or a ratio of <0.1, when the theoretical value was greater than or equal to 100. This step removed 15–30% of the peptides per run. The second filter was applied at the protein level. Peptides from a single technical replicate corresponding to a single protein were pooled. Those proteins not represented in at least two biological replicates were dropped. Lastly, peptides not present in at least two biological replicates were dropped and the resulting single peptide identifications were dropped. The reduced Sequest results, as well as the Mascot results were compiled in Scaffold (version 2.02.01, Proteome Software Inc., Portland, OR) in order to validate MS/MS based peptide and protein identification. Peptide identifications were accepted if they could be established at greater than 90% probability as specified by the Peptide Prophet algorithm [18]. Protein identifications were only accepted if they could be established at greater than 90% probability and contained at least two identified peptides. Protein probabilities were assigned by the Protein Prophet algorithm [19]. At this stage, all remaining peptide identifications were screened for presence in multiple biological replicates and those proteins identified by two peptides were subject to manual validation for final confirmation. The final, compressed files and accepted spectra are available for review through Tranche (https://proteomecommons.org/tranche/; entitled “30day_mudpitted_condensed” and “90day_mudpitted_condensed”).

### Normalization of Spectral Counts

In order to compare the two time-points, the spectral counts were normalized. To do this, the average number of spectra in both 30 and 90-day analysis (321,627) was divided by the 30-day total (355,411) and the 90-day-total (287,843) yielding the values 0.9 and 1.12, which were used as multipliers to the spectral counts in the 30 and 90-day samples respectively [68].

### Calculation of False Discovery Rates (FDRs)

In order to calculate the FDR for each of the time-point data sets, all .raw files were searched against a decoy database. Decoy databases contain a reversed version of the proteins included in either the customized database composed from the *Mycobacterium tuberculosis* H37Rv FASTA (for Sequest), as well as concatenated to the reverse IPI Mouse database (for Mascot). The resulting files were pooled in Scaffold and the analysis was identical to that described above. The number of proteins matched to the reverse database, 35 and 23, in the 30 and 90-day data sets respectively, is equal to the number of false positives (FP). The equation for the FDR is FP/TP, where TP is the number of total positives found in each data set (318 for day 30 and 334 for day 90). Therefore, the FDRs are 11% and 6.8% for the 30 and 90-day data sets, respectively. In addition, uninfected guinea pig lungs were subjected to the identical protocol including lung homogenization and process through mass spectrometry and analysis. This analysis identified 74 *Mtb* proteins, however only 11 of these proteins contained peptides identified in our 30 and 90-day infected lung samples. These 11 proteins were considered false positives and removed from the analysis of infected tissues. Of the remaining 63 “*Mtb* proteins” (Supplementary Table S4, Supplementary Figure S1A), 29 and 23 were identified in the 30 and 90-day infected lung samples, respectively. However, none of the peptides used to make these identifications were identical to those found in infected lung tissue, thus, these proteins were retained in our study (Supplementary Figure S1B). By retaining these proteins, an FDR of 9.1% and 6.9% for day 30 and 90-day samples, respectively, is derived. This rate is compares with the FDR calculated by decoy method described above, supporting the traditional statistical validation used.

### Western Blots

For validation of proteins, two antibodies against *Mtb* proteins identified in this study, anti-GlcB and anti-PstS1, were available and provided through the NIAID contract, “Tuberculosis research materials and vaccine testing” (HHSN266200400091c). 10 µg of each lung homogenate sample and the ECL Plex Fluorescent Rainbow Marker (GE) Healthcare) were resolved by SDS-PAGE on a 4 – 12% Bis-Tris Mini Gel (Invitrogen) in MES Buffer, followed by transfer to a PVDF membrane (Hybond LFP, GE Healthcare). Western blot procedure was performed as recommended by the manufacturer. ECL Plex Cy2 conjugated anti-mouse IgG was used as the secondary antibody. Blots were scanned using the Typhoon scanner (GE Healthcare), and bands were analyzed using ImageQuant™ TL software. *Mtb* whole cell lysate (2.5 µg) was used as the positive control (Supplementary Figure S2).

## Supporting Information

Table S1 Mtb proteins identified in 30-day infected guinea pig lungs. Data includes number of unique peptides identified for each protein.
(0.10 MB XLS)

Table S2 Mtb proteins identified in 90-day infected guinea pig lungs. Data includes number of unique peptides identified for each protein.
(0.10 MB XLS)

Table S3 Spectral counts of the Mtb proteins identified in 30-day and 90-day infected guinea pig lungs.
(0.22 MB XLS)

Table S4 Spectral Counts of the Mtb Proteins Identified in Uninfected Guinea Pig Lungs.
(2.57 MB XLS)

Figure S1 (0.30 MB TIF)

Figure S2 Fluorescent western blots of GlcB (80.4 kDa) and PstS1 (38.2 kDa) at 30 and 90 day infection time points. C: positive control; U: uninfected guinea pig; 1-6: infected guinea pigs.
(0.26 MB TIF)
