# Supplementary figures and images for: Portrait of a Pathogen: The Mycobacterium tuberculosis Proteome In Vivo
Source: PLoS One. 2010 Nov 11;5(11):e13938. doi: 10.1371/journal.pone.0013938 (PMC2978697; doi:10.1371/journal.pone.0013938)

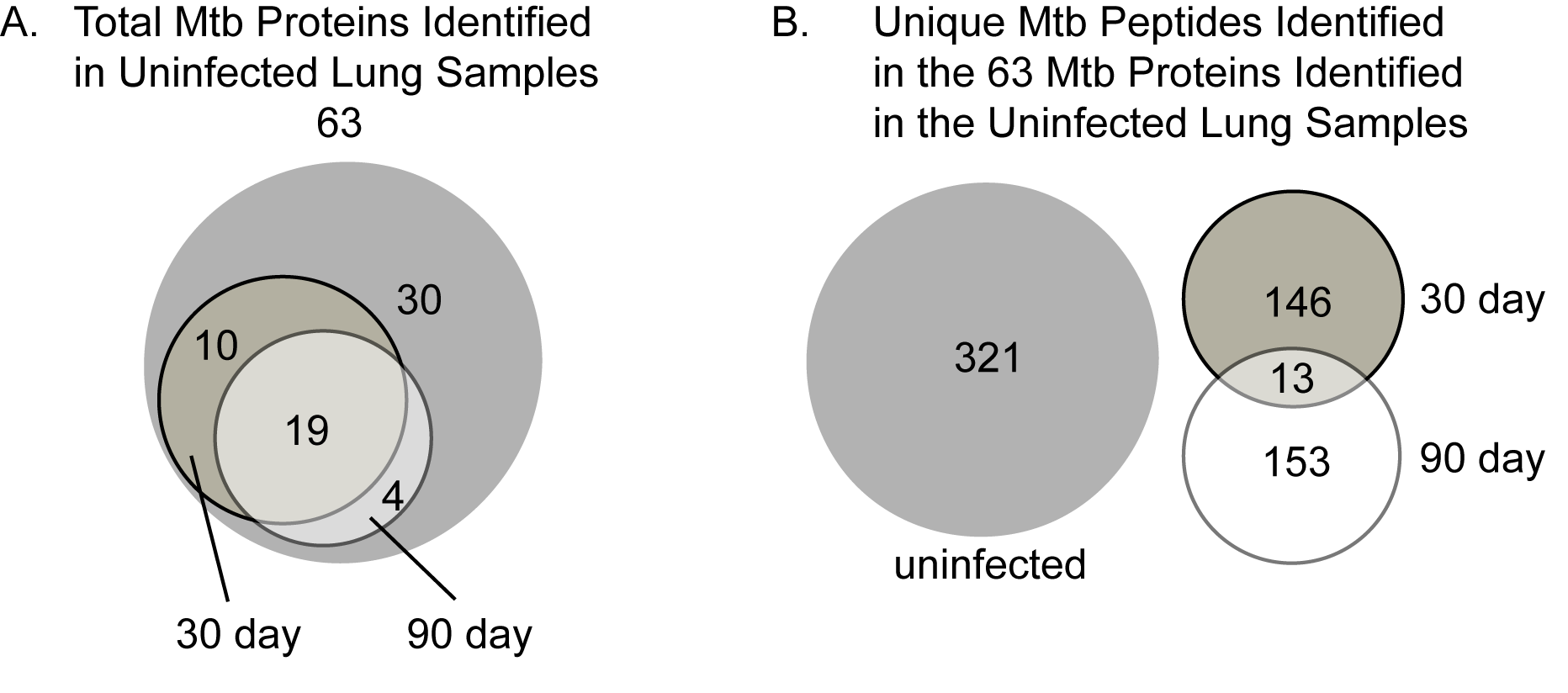

Supplement: Figure S1 — (0.30 MB TIF) [file pone.0013938.s005.tif]

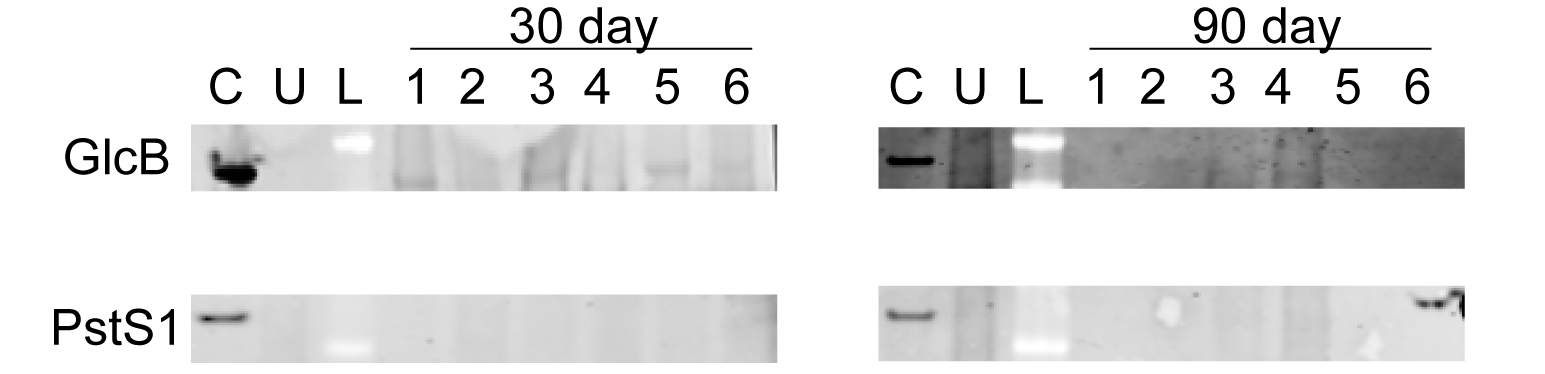

Supplement: Figure S2 — Fluorescent western blots of GlcB (80.4 kDa) and PstS1 (38.2 kDa) at 30 and 90 day infection time points. C: positive control; U: uninfected guinea pig; 1-6: infected guinea pigs. (0.26 MB TIF) [file pone.0013938.s006.tif]
